# Innovative Intergenerational Initiatives

**DOI:** 10.1093/geroni/igaf122.875

**Published:** 2025-12-31

**Authors:** Tamar Shovali, Lisa Borrero

## Abstract

This session highlights lessons learned, strategies for success, and setbacks to avoid when developing innovative and collaborative intergenerational initiatives. The first presentation examines the process and outcomes of a series of course-based intergenerational Death Cafes that brought together students, community members, and members of a university-affiliated Academy of Senior Professionals to discuss death and dying. The second presentation shares collaborative strategies for forming an interdisciplinary team to develop a 100th day of school evidence-based toolkit for pre-K-2 teachers to foster intergenerational understanding from an early age. The third presentation features the Sages and Scholars innovative program to promote age inclusivity that creates shared learning experiences between students and a university-affiliated Osher Lifelong Learning Institute. The fourth presentation explores bringing traditional students and older learners together to create, express, and learn together in a course titled Intergenerational Artistic Expression. The final presentation highlights an innovative model for intergenerational advocacy and partnership through the Intergenerational Community Health Collaborative, a program that connects residential older adults with students to discuss pressing community health issues. In addition to presenting multiple intergenerational initiatives using a variety of innovative approaches, this symposium session highlights the creativity and dynamism that intergenerational innovations can provide to foster meaningful connections. These presentations demonstrate expanding horizons to create inclusive spaces that foster contributions of people of all ages, encourage the bi-directional exchange of life experiences, and inspire innovative approaches to promoting connectedness across our increasingly diverse society. Intergenerational Learning, Research, and Community Engagement Interest Group Sponsored Symposium

